# Prevalence and Risk Factors of Severe Dry Eye in Bangladesh-Based Factory Garment Workers

**DOI:** 10.3390/diagnostics10090634

**Published:** 2020-08-26

**Authors:** Mamunur AKM Rashid, Calesta Hui Yi Teo, Sumaiya Mamun, Hon Shing Ong, Louis Tong

**Affiliations:** 1 Al Noor Eye Hospital, 1/9 E, Satmasjid Road, Lalmatia, Dhaka 1207, Bangladesh; mamun3312@gmail.com; 2 Singapore Eye Research Institute, The Academia, 20 College Road, Disovery Tower Level 6, Singapore 169856, Singapore; teohuiyicalesta@gmail.com (C.H.Y.T.); honshing@gmail.com (H.S.O.); 3 Institute of Nutrition & Food Science, University of Dhaka, Dhaka 1000, Bangladesh; sumaiya.mamun@du.ac.bd; 4 Singapore National Eye Centre, 11 Third Hospital Ave, Singapore 168751, Singapore; 5 Duke-NUS Medical School, 8 College Rd, Singapore 169857, Singapore; 6 Yong Loo Lin School of Medicine, National University of Singapore, 10 Medical Drive, Singapore 117597, Singapore

**Keywords:** dry eye, meibomian gland dysfunction, ocular surface disease, Bangladesh, global health, epidemiology, public health, patient reported outcome measures

## Abstract

This study sought to evaluate the prevalence of dry eye and meibomian gland dysfunction (MGD) and the associated factors of severe dry eye symptoms (SDES) among garments worker of Gazipur, Bangladesh. We prospectively collected cross-sectional data for 1050 garments workers of a factory (70% response). All participants had an evaluation of the Ocular Surface Disease Index (OSDI), and a detailed ophthalmic examination including tear breakup time (TBUT), ocular surface fluorescein staining, and Schirmer’s I test. MGD grading was based on the viscosity/color and ease of manual expression of meibum. Mean age of participants was 35.5 ± 12.1 years; 53.8% were women. The prevalence of dry eye (OSDI > 12) was 64.2% (95% CI 61.2–67.1%). OSDI was not significantly different between sex or age-groups but associated with increasing MGD grade (*p* < 0.001), reduced TBUT (<5 s) [*p* < 0.001], and reduced Schirmer’s test (<5 mm) [*p* < 0.001]. Thirty-five percent had SDES (OSDI > 32). Using univariate logistic regressions, SDES was associated with older age (Odds Ratio (OR) 1.01, 95% Confidence Interval [1.005–1.03] per year increase) and male sex (OR 1.76, 95% CI: 1.36–2.27). When adjusted for age and sex, SDES were strongly associated with increase in MGD severity grading (OR 188, 95% CI: 91–390). However, in multivariate regression, TBUT, but not MGD severity, became the only significant determinant of SDES (OR 13.0, 95% CI: 6.3–27.0, for every 1 s decrease in TBUT). MGD is common in garments workers, contributing to dry eye symptoms in addition to other tear parameters. Reduced tear stability is associated with SDES.

## 1. Introduction

Dry Eye Disease (DED), a multifactorial disease of the ocular surface and loss of homeostasis of the tear film, is associated with visual disturbance, symptoms of ocular discomfort, and tear instability [1,2,3]. Tear film & Ocular Surface Society Dry Eye Workshop (TFOS DEWS) II reported that this disease affects about 5–50% of the population. The large variation is due to a large number of research studies on small geographically homogeneous populations. The epidemiology sub-committee emphasized the need to expand prevalence studies to more geographical regions, and to include different races and ethnicities [4]. Dry eye can have a significant impact in patients’ visual function and quality of life, adversely hindering the ability to carry out daily activities, such as reading or driving. This disease of the ocular surface has thus been an increasing public health concern and as it poses significant socioeconomic implications [5,6,7,8,9].

Blepharitis and meibomian gland dysfunction (MGD) are major associated factors of DED. MGD is characterized by chronic abnormalities of the meibomian glands, resulting in altered meibum delivery to the tear film, which can result in poor tear film stability or poor breakup times, a type of dry eye classified as evaporative dry eye. On the other hand, dry eye may also be due to aqueous tear deficiency [10,11].

There are scarce data on the epidemiology of dry eye in developing countries within Asia. In developing countries, dry eye has received minimal clinical management and investigative attention compared to other eye diseases. The healthcare burden of dry eye in such countries is essentially unknown [12]. In Indonesia, the age adjusted prevalence of dry eye symptoms is 27.5% (95% CI: 24.8–32.2), and current smoking and pterygium were found to be independent risk factors for the DED [13]. In Palestine, older age and female gender (both *p* =  0.001) were significantly associated with DED [14].

Bangladesh, the 8th most populous country in the world, is located in South Asia between India and Myanmar, occupying an area of 57,000 square miles. The per capita GDP of Bangladesh is US$4992; though classified as a low-income country, it has the fastest growing real GDP in the world. With a population of 163 million, a properly designed epidemiological study will elicit risk factors and knowledge on dry eye that may not be possible in smaller studies elsewhere.

Ocular surface disease has been shown to represent up to a quarter of eye diseases in the urban slums of Dhaka, Bangladesh. A population-based study here found a prevalence of 3.2% for DED, 1.4% for blepharitis, 17.1% for conjunctivitis, and 3.0% for pterygium. This study had limitations in its methodology since slit-lamp examination was only used in the study, and symptoms were not quantified [15]. We believe that the reported figures are underestimated, because the prevalence rates of conditions like DED and blepharitis are known to be higher when clinical symptoms are included in their definitions [13,16,17,18,19,20,21].

There have not been any other large community-based studies of DED from Bangladesh. As DED is affected by socioeconomics, lifestyle, occupations, and environmental factors, it is likely to have different associated factors in developing countries, such as Bangladesh, compared to developed countries. For instance, there may be more DED in cases of trachoma and vitamin A deficiency, and in special occupational groups [22,23,24].

The severity of ocular surface symptoms can be quantified using an internationally recognized and validated questionnaire, such as the Ocular Surface Disease Index (OSDI) [2,25,26,27,28]. Several studies have described the relationship of OSDI scores and clinical signs, but these are primarily hospital-based studies [4,29,30,31,32,33].

In this study, we performed a community-based study in a group of factory garment workers in Bangladesh, to investigate the prevalence of DED and MGD, the associated risk factors of severe dry eye symptoms (SDES), and the dry eye symptoms in different subtypes of DED (tear instability/aqueous tear deficiency).

## 2. Methods

### 2.1. Study Design

This is a prospective cross-sectional study conducted in a single garment factory in the town of Gazipur, Bangladesh. The study involves only one visit to the Ophthalmologist.

### 2.2. Participants

Participants had given informed verbal consent. The study obtained approval from the Bangladesh Medical Research Council (BMCR) institutional review board (BMRC/NREC/2017-2018/1157, approved on 2 August 2018), and only utilized clinically accepted procedures and complied with the Tenets of Declaration of Helsinki for human research.

### 2.3. Inclusion and Exclusion Criteria

We excluded garment workers with red eyes, ocular surgery done in the last six months and those working in air-conditioned or separate rooms. We did not evaluate for use of chronic medications.

### 2.4. Dry Eye Diagnosis

We calculated the prevalence of dry eye based on presence of symptoms with one or two clinical signs in accordance to the Tear film & Ocular Surface Society Dry Eye Workshop (TFOS DEWS) II Diagnostics Report. “Dry eye is a multifactorial disease of the ocular surface characterized by a loss of homeostasis of the tear film, and accompanied by ocular symptoms, in which tear film instability and hyperosmolarity, ocular surface inflammation and damage, and neurosensory abnormalities play etiological roles. If a patient has dry eye symptoms, DED is diagnosed when at least one homeostasis test result is positive.” [2].

### 2.5. Study Procedures

All participants underwent the following clinical procedures on the initial referral visit.

#### 2.5.1. Questionnaire

All participants underwent a symptom evaluation using the Ocular Surface Disease Index (OSDI^©^, Allergan, Inc., Irvine, CA, USA). Briefly, the OSDI questionnaire consisted of 12 questions, each question graded from 0 to 4. The total OSDI scores on the scale of 0 to 100 were then calculated with the OSDI^©^ (Allergan, Inc., Irvine, CA, USA) formula (sum of scores) × 25/(12 questions), with higher scores representing greater symptoms severity [25].

#### 2.5.2. Fluorescein Breakup Time (TBUT)

Briefly, a minimally wet (saline) fluorescein strip was used to instill fluorescein dye. The fluorescein tear breakup time (TBUT) is performed as in a previous study [34,35]. A drop of normal saline was applied to the Florets (fluorescein strip) and shaken dry. The tip of the strip was then applied on to the participant’s lower fornix. After blinking three times, the participant was asked to keep the eye closed. On opening the eye, the participant was asked to keep the eye opened for as long as possible, staring at the examiner’s forehead. The TBUT was taken as the interval from the time of opening of the eyelid to the appearance of the first dry spot on the cornea, as observed on the slit-lamp biomicroscope. The measurement was obtained to the nearest second and the average of three times. The procedure for this step has been previously described [36,37].

#### 2.5.3. Schirmer‘s I Test

The Schirmer‘s I test (without prior anesthesia) was done with standard 5 mm wide test strips (Clement Clark^®^, Essex, UK) with a notch for folding. Briefly, the schirmer strip was folded at the notch and applied over the middle to lateral one third of the inferior eyelid, with the rounded end of the strip in the inferior fornix. Care was taken not to induce reflex tearing. The test was applied for five minutes and the reading in mm was recorded as the length of wetting of the strip from the notch. The Schirmer‘s I test was performed as in previous publications [34,36].

#### 2.5.4. Meibomian Gland Dysfunction (MGD) Examination

The characteristics of the meibum secreted was evaluated by one ophthalmologist using slit-lamp microscopy and graded as follows: 0: clear meibum, 1: colored meibum with normal consistency, 2: viscous meibum, 3: inspissated meibum, and 4: blocked meibomian gland. This was used as a measure of MGD severity.

#### 2.5.5. Slit-Lamp Examination

Other clinical features were examined using a slit lamp biomicroscope. This included scurfing/crusting, subtarsal papillary reaction [36,38] and regularity of the eyelid margin [39]. The presence of scurfing/crusting and regularity of the eyelid margin was performed subjectively by one single ophthalmologist at the slit lamp biomicroscope. This was recorded as a dichotomous variable “0“ absent, or “1“ present. Excessive powder or flakes on the eyelashes would be considered as significant. In the case of the eyelid margin, irregularity was observed in cases with significant eyelid scarring and fibrosis, or rounding of the eyelid margin. In the upper eyelid, subtarsal conjunctival papillary grading was performed after eversion of the upper eyelid. The tarsal plate was observed under the slit lamp biomicroscope and graded in an ordinal scale from 0–3 using the CCLRU grading system [39]. The lower eyelid papillary grading was similarly assessed and graded by pulling down the lower eyelid, rotating it to expose the inferior fornix. Corneal sensitivity was also screened using a cotton wisp [40].

### 2.6. Statistical Analysis

Statistical analysis was performed using StataCorp. 2013. Stata Statistical Software: Release 13.1. College Station, TX, USA: StataCorp LP.

The frequency distribution of the OSDI was plotted as a histogram and the profile examined for normality. Deviation from normality was evaluated with the Shapiro–Wilk test and with observation of the histogram curve.

Continuous variables (TBUT, Schirmer’s test) were then categorized into two categories to more than or less than a threshold. These two categories would form the basis of evaluating OSDI as an outcome using the *t* test. For example, in the case of TBUT, participants were divided into those with TBUT less than five seconds, and those with TBUT 5 or more seconds since this is a commonly used threshold. The OSDI of the participants in these two groups were then compared using the *t* test.

Similarly, the OSDI between male and female sex participants was compared using the *t* test.

For ordinal variables with multiple values (e.g., the MGD grading based on number of liquid meibum expressing glands), the OSDI was analyzed using ANOVA.

Multivariate regression was performed with OSDI as the dependent variable. We performed models using only the clinical signs and demographics of patients, as well as models introducing the predisposing factors of dry eye, such as concomitant drugs and medical conditions.

Logistic regression was performed to examine the relationship of the various significant factors with OSDI as a dichotomous variable (OSDI) (severe: >32; moderate and below: ≤32). The scoring thresholds for OSDI were previously published [25,41].

The subtypes of dry eye (pure evaporative, pure aqueous deficiency, mixed) were defined based on TBUT and Schirmer’s test findings as explained below.

Statistical significance was based on alpha of 0.05.

We did not calculate the sample size required to estimate the prevalence of dry eye, because we utilize a 100% sampling of the factory. We recognize that this may not be representative of all garment factories in Bangladesh.

## 3. Results

### 3.1. Clinical and Characteristics of Participants

**Figure 1:**
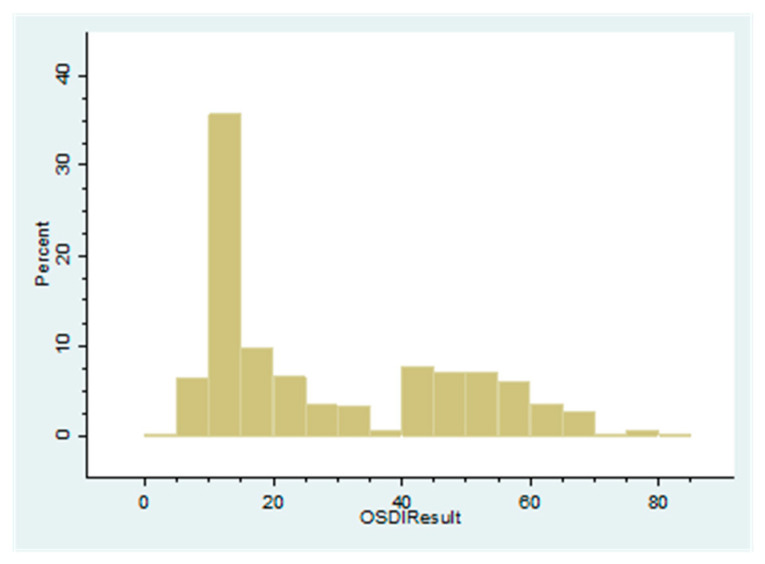
Histogram of Ocular Surface Disease Index (OSDI) values in factory workers.

In this study, mean age of participants was 35.56 ± 12.12 years (range 18 to 59). Fifty-three point eight percent were women. Sixty-four point two percent (95% CI: 61.2–67.1) of participants had dry eye defined as OSDI > 12. However, if the definition also required a clinical sign to be abnormal, the prevalence would be between 35 and 40% (Table 1). The OSDI followed a bimodal distribution (Figure 1) with highest peak at 10–15 and another at 40–45 years. Only 46 (4.4%) participants are supervisors which had a higher socioeconomic group (higher monthly income), but this small proportion prevented further analysis of the effect of socioeconomic status on the outcomes of interest below.

### 3.2. Meibomian Gland Dysfunction

**Figure 2:**
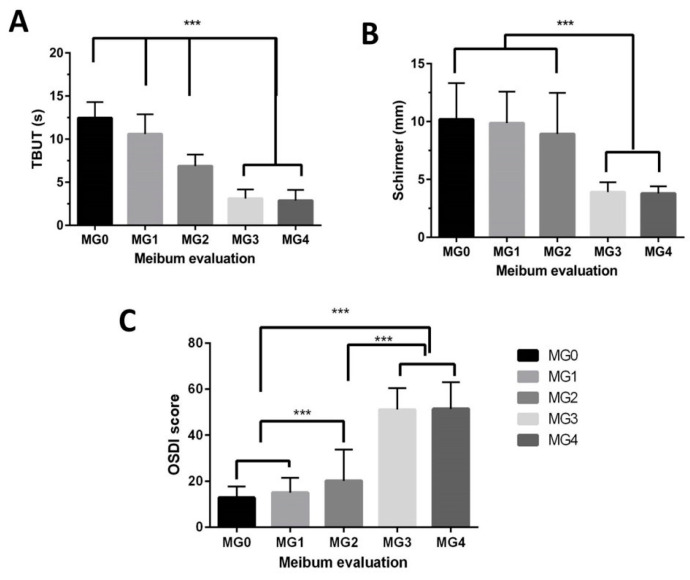
Relationship of meibomian gland dysfunction (MGD) grading with (**A**) tear breakup times (TBUT), (**B**) Schirmer’s I, and (**C**) Ocular surface disease index (OSDI). The higher the OSDI, the greater the severity of dry eye symptoms. MG0: clear meibum, MG1: colored meibum with normal consistency, MG2: viscous meibum, MG3: inspissated meibum, and MG4: blocked meibomian gland. *** *p* ≤ 0.001; error bar = standard deviation

The proportions of participants with grades 0, 1, 2, 3, and 4 were 26.2% (275/1050), 17.3% (182/1050), 23.3% (245/1050), 28.6% (300/1050), and 4.6% (48/1050), respectively. The relationship between MGD and the other signs of dry eye (Schirmer’s and TBUT) is shown in Figure 2A,B.

### 3.3. Factors Affecting OSDI

The OSDI increased with increasing MGD severity grading (*p* < 0.001) and was associated with lower TBUT (<5 s) [*p* < 0.001), and lower Schirmer’s test (<5 mm) [*p* < 0.001). Using multiple linear regression (Table 2), OSDI was significantly and independently associated with higher meibomian gland (MG) grading, lower values of Schirmer test and TBUT (all *p* < 0.001). The relationship between OSDI and MGD is shown in Figure 2C.

### 3.4. Multiple Linear Regression of the Factors Affecting OSDI

We performed several diagnostics to evaluate the validity of the multiple regression.

**Figure 3:**
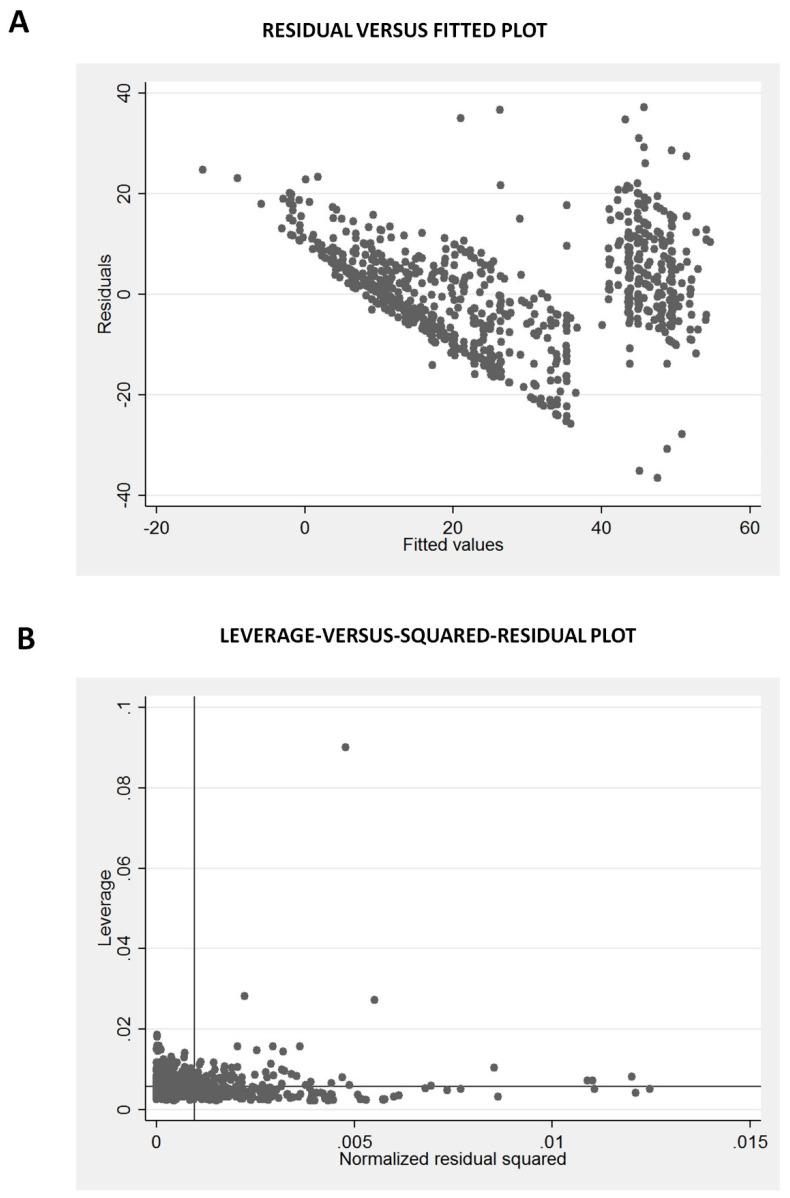
Graphs showing the residual versus fitted plot (**A**) and the leverage versus squared residual plot (**B**).

**Figure 4:**
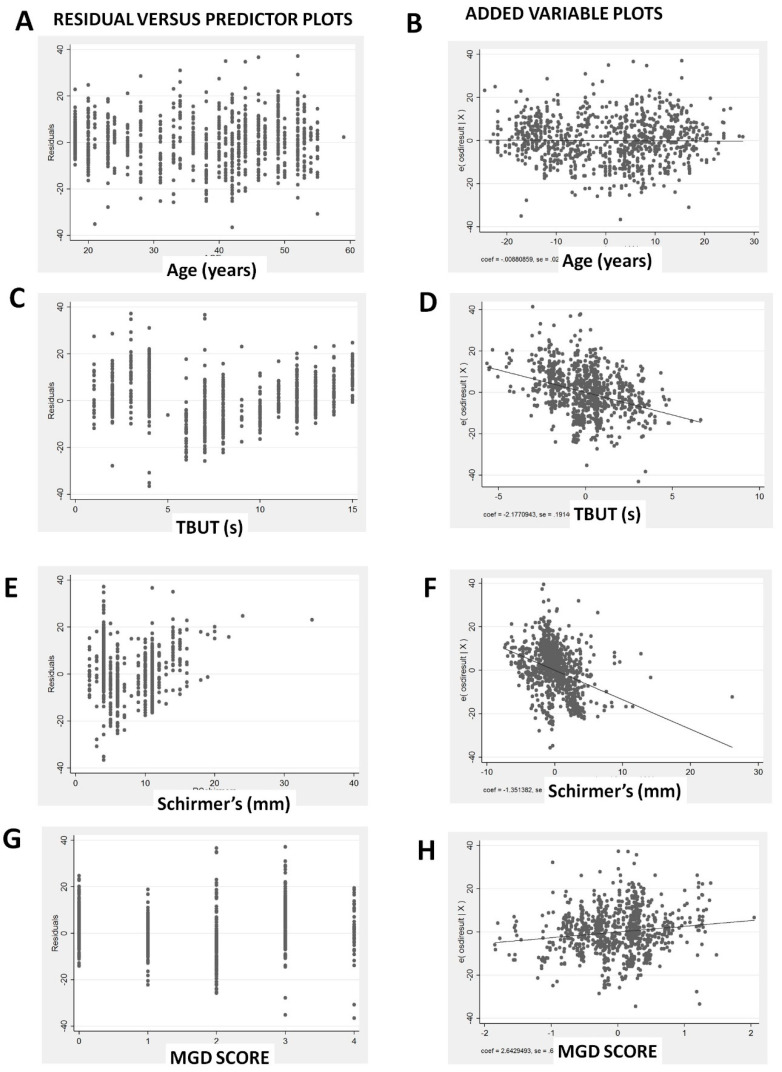
Graphs showing the residual versus predictor plots (**A**,**C**,**E**,**G**) and the added variable plots (**B**,**D**,**F**,**H**).

**Figure 5:**
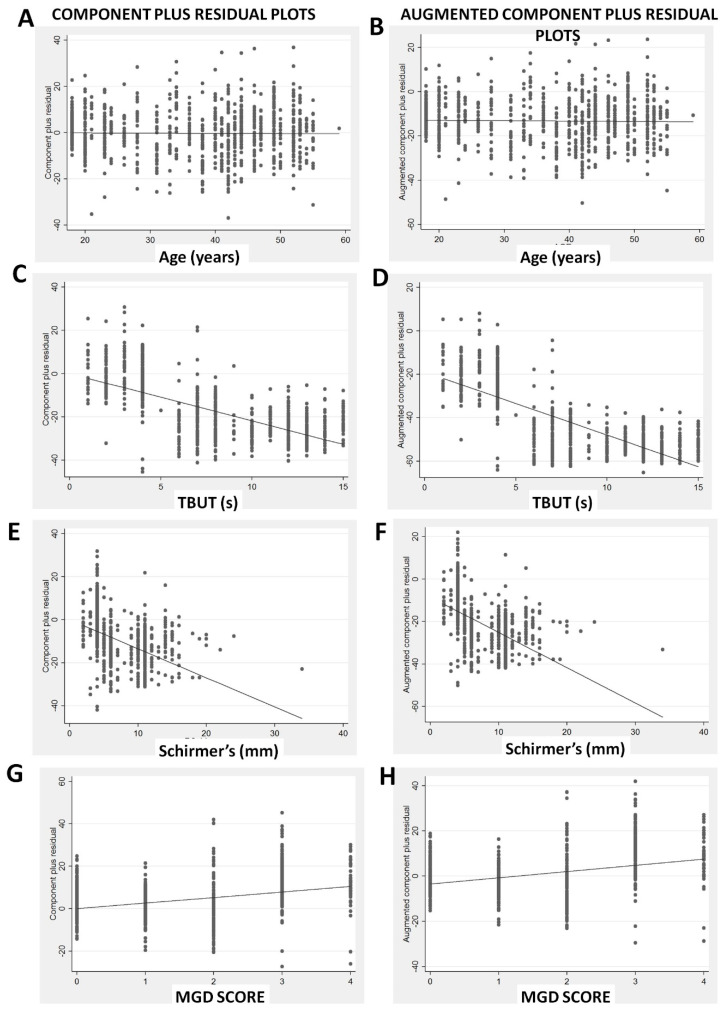
Graphs showing the component plus residual plots (**A**,**C**,**E**,**G**) and the augmented component plus residual plots (**B**,**D**,**F**,**H**).

In the residual versus fitted plot (Figure 3A), there is non-linearity of the residuals beyond the fitted values of more than 40, suggesting a violation of the assumption that OSDI is linear in our independent variables. There is also increased variation of the residuals above fitted values of 40, suggesting heteroscedasticity, and some violation of the least squares assumptions. However, it is reassuring that the leverage versus squared residual plot (Figure 3B) shows no points with excessively high leverage given the squared residuals. The residual versus predictor plots (Figure 4A,C,E,G) did not find any marked violations of the regression assumptions except that the residuals are reduced with TBUT of 5–10 s (Figure 4C), and the variability of the residuals is reduced for Schirmer’s from 10 to 20 mm (Figure 4D). The added variable plots (Figure 4B,D,F,H), also called partial regression leverage plots or adjusted partial residual plot, show only one notable outlier in Schirmer’s (Figure 4F, one case in right lower quadrant). The component plus residual plots (Figure 5A,C,E,G) and augmented component plus residual plots (Figure 5B,D,F,H) show no significant non-linearity of the independent variables, except perhaps a flattening for TBUT above 5 s (Figure 5C,D). Hence, overall, the multiple regression model is reasonable and has only a few minor violations.

### 3.5. Risk Factors for Severe OSDI

The proportions of severe dry eye symptoms (SDES) (OSDI > 32) by age, sex, Schirmer’s test, and TBUT, is shown in Table 3. Thirty-five percent of the participants had SDES (defined as OSDI > 32). Using univariate analyses, severe OSDI is associated with male, Schirmer’s < 5 mm and TBUT < 5 s (*p* < 0.001).

Using univariate logistic regressions, SDES was associated with age (Odds Ratio (OR) 1.01, 95% Confidence Interval (1.005–1.03) per year increase) or male sex (OR 1.76, 95% CI: 1.36–2.27). When adjusted for age and sex, SDES were strongly associated with increase in MGD severity grading (OR 188, 95% CI: 91–390). However, MGD severity was no longer significant in a multivariate logistic regression model, and TBUT became the only significant determinant of severe dry eye (OR 13.0, 95% CI: 6.3–27.0, for every 1 s decrease in TBUT). The association remained significant even after adjustment for demographic factors and Schirmer’s (Table 4). This suggests that the mechanism of MGD influencing OSDI is through a reduction of tear stability (lowered TBUT).

Interestingly, a low TBUT is near ubiquitous in participants with SDES (Table 5).

### 3.6. Subtypes of Dry Eye

In this study, 414/1050 (39.4%, 95% confidence interval: 36.5–42.5) participants had abnormality in at least one of the two tests (Schirmer’s and TBUT). The mixed subtype of dry eye was most common, with 368 participants having mixed dry eye (Schirmer’s test ≤5 mm, TBUT ≤5 s), 45 participants had pure aqueous deficiency (Schirmer’s test ≤5 mm and TBUT >5 s), and one participant had pure evaporative dry eye (Schirmer’s test >5 mm and TBUT ≤5 s).

The OSDI was significantly worse in the mixed type of dry eye compared to pure aqueous deficient type (*p* < 0.001), with OSDI of 51.3 ± 9.4 and 15.4 ± 7.7, respectively.

The clinical severity of the MGD was greater in the mixed type of dry eye (3.1 ± 0.4) compared to the pure aqueous deficient type (1.0 ± 0.9) (*p* < 0.001). The age of the participants with these two subtypes of dry eye was similar. There was only one case of pure evaporative dry eye, so further analysis cannot be performed. Unlike many populations, the pure evaporative type of dry eye was uncommon in our study population.

## 4. Discussion

In this community study of a single occupational group of garment workers, we found a prevalence of DED of 64.2% (defined by only symptoms), 35–40% (symptoms in combination with signs). These prevalence rates are higher compared to general population-based studies previously reported in other countries, [17,19,42,43] although symptoms were not evaluated using OSDI in many of these previous studies. We found that the OSDI significantly increased with higher MGD grades, and was associated with reduced TBUT (<5 s) and reduced Schirmer’s tests (<5 mm). Reduction of TBUT was the major associated factor for SDES. We also found that mixed subtype of aqueous tear deficiency and evaporative DED was the most common subtype in our study population; patients in this subtype also showed the most symptoms compared to other subtypes.

### 4.1. Previous Studies

Although there were no reported studies of ocular surface diseases conducted on garment factory workers, studies have reported DED in other occupational environments, such as cleanrooms [22,23] and in a chemical factory [24]. In a study investigating 91 cleanroom workers exposed to insufficient or flashing lights, 41.8% of the participants had symptoms of DED and 63.7% had symptoms of eye fatigue [22]. In another three-year study involving cleanroom workers, the TBUT and McMonnies questionnaire results worsened with more years of employment [23]. In a tertiary hospital-based study in rural India, ten participants were factory workers, and nine were found to have DED, but the type of the factory was not reported [44]. A pilot study carried out on 78 workers in a chemical factory has found that the TBUT for workers exposed to plastic and paint intermediates (6 ± 4.84 s) were significantly (*p* < 0.012) lower than non-exposed workers (11 ± 4.80 s). Some chemicals in this factory include formaldehydes which were also found in clothing [24].

A previous cross-sectional study done on 2346 newly referred dry eye patients from the Singapore National Eye Centre, showed the severity of dry eye symptoms measured by OSDI were contributed mainly by a reduction in expressible meibomian glands [34]. While there are no population-based studies that had previously linked OSDI with an increase in MGD, our findings were consistent with a previous clinic-based study (*n* = 176) in Beijing, China. In this study, OSDI was found to be weakly but significantly correlated with several MGD signs: number of MG orifice in central 1 cm of upper eyelids (r = 0.359, *p* < 0.001), plugging of orifices (r = 0.281, *p* < 0.001), and meibum properties (r = 0.172, *p* < 0.001). Multiple linear regression analyses show that OSDI was significantly associated with the nine clinical signs of MGD (including lid margin hyperemia and plugging of MG orifices) and with reduced TBUT [45].

Two other studies, which did not measure OSDI, were consistent with our results. In the first study, involving 201 patients in Tokyo Japan, 22.4% of the patients with ocular discomfort either had an obstruction of an orifice or gland dropout, or both, whereas, in those without ocular discomfort, only 9.3% of these patients had the MGD signs (*p* = 0.03) [46]. In a second study conducted on 63 eyes in Peking University Hospital, increase in two of the ten specific dry eye symptoms, foreign body sensation (r = 0.265, *p* = 0.041), and blurring of vision (r = 0.390, *p* = 0.030) were found to be weakly associated with a reduced expressibility of the MGs [47].

Furthermore, in a case-controlled study performed by our group using the Standard Patient Evaluation of Eye Dryness (SPEED) questionnaire, we found that increased symptoms of dry eye and irritation was associated with reduced expressibility of MGs [48].

### 4.2. Proposed Mechanism

Chemicals are found in the clothing-making process. Phthalates have been used as plasticizers, or as softening agents in polyvinyl-chloride (PVC). PVC is also used in decorative printing on garments and T-shirts [49].Chronic exposure to other chemicals used in textile production, such as benzothiazole or derivatives [50] and fabric dyes [51], are reported to induce allergies in other mucosa, such as the skin [52]. Low grade allergies can also contribute to ‘dry eye-like’ symptoms in the eye. In addition, the indoor environment of a factory, such as high temperature, humidity, ventilation, and air distribution, may have a deleterious effect on the pre-corneal tear film [53].

In MGD, delivery of abnormal meibum contributed to a reduction in the function of the tear lipid layer, which may reduce tear stability. Due to lack of protection of the ocular surface, reduced tear stability leads to stimulation of corneal nerves. In addition, reduced tear stability may damage the ocular surface and result in the production of inflammatory mediators that stimulate corneal nerves, contributing to increased dry eye symptoms [54,55,56,57,58,59].

### 4.3. Strengths & Weaknesses

This study involved a large sample in the community within a single occupational group.

The use of a uniform protocol by a single ophthalmologist provided an accurate and comprehensive analysis and with a higher confidence of estimates. Since the study did not employ a standard force MG evaluator, the number of MGs yielding liquid meibum was not documented. 

Minimally invasive techniques may be preferred for the examination of the ocular surface, because an invasive technique can interfere with the tear film to the extent that subsequent tests become unreliable. In this study we have included a less invasive strip meniscometry procedure and will report that in a separate paper. Fourier-domain optical coherence tomography may be used to evaluate tear film dynamics and epithelial changes under physiologic and pathologic conditions [60]. One must also establish the validity of using the less invasive tests before drawing conclusions, since these are generally less investigated in previous epidemiological studies.

### 4.4. Conclusion, Clinical Implications and Future Direction

DED and MGD are common amongst Bangladesh garment factory workers. We found that SDES were found mainly in males and those with lower TBUT. Furthermore, MGD and reduced tear stability play important roles in dry eye symptoms. The mixed type of DED is most common, with significantly worse symptoms in this subgroup.

Our results suggest that occupational screening for DED may be warranted in certain types of occupations in Bangladesh. MGD, resulting in reduced TBUT, contributes to the morbidity of the patient. Here, our results suggest that more attention should be paid to underlying MGD and tear instability in terms of its documentation and treatment. Documentation of MG activity should be quantified wherever possible. The use of a standard force MG evaluator may be advantageous for clinical and epidemiological assessment of MG function. Emerging treatments for MGD may play a bigger role in people in certain occupational groups who suffer from higher risk of dry eye [61,62,63,64].

## Figures and Tables

**Table 1:** Prevalence of dry eye in factory workers in Bangladesh.

*n* = 1050	OSDI > 12 and Two Signs Abnormal (TBUT < 5 s and Schirmer < 5 mm)	OSDI > 12 and at Least One Sign Abnormal (TBUT < 5 s, Schirmer < 5 mm)	OSDI > 32 and Two Signs Abnormal (TBUT < 5 s and Schirmer < 5 mm)	OSDI > 32 and at Least One Sign Abnormal (TBUT < 5 s, Schirmer < 5 mm)
Prevalence (n)% [95% confidence interval]	(367) 35.0 [32.1–37.9]	(414) 39.4 [36.5–42.5]	(364) 34.7 [31.8–37.6]	(414) 39.4 [36.5–42.5]

**Table 2:** Multiple regression with OSDI score as the dependent variable.

Independent Variables	Coefficient	Standard Error	*p* Value
Age	−0.01	0.03	0.75
Sex ^Ϯ^	1.28	0.65	0.05
Tear breakup time	−2.18	0.19	<0.001
Schirmer’s test	−1.35	0.12	<0.001
MG grading	2.64	0.61	<0.001

^Ϯ^ Sex: Female = 1, Male = 2.

**Table 3:** Distribution of severe dry eye (OSDI > 32) by different factors.

		OSDI Group		*p* Value
		<32	>32	
**Total**		376 (35.8%)	674 (64.2%)	
**Sex**	Male	141 (37.5%)	344 (51.0%)	<0.001
	Female	235 (62.5%)	330 (49.0%)	
**Age Group**	18 to 30	161 (42.8%)	210 (31.2%)	<0.001
	31 to 40	76 (20.2%)	143 (21.2%)	
	41 to 50	97 (25.8%)	250 (37.1%)	
	>50	42 (11.2%)	71 (10.5%)	
**Schirmer’s**	Normal > 5	352 (93.6%)	285 (42.3%)	<0.001
	Dry Eye < 5	24 (6.4%)	389 (57.7%)	
**TBUT**	Normal > 5	374 (99.5%)	307 (45.5%)	<0.001
	Dry Eye < 5	2 (0.5%)	367 (54.5%)	

**Table 4:** Logistic regression with severe dry eye.

Independent Variables	Odds Ratio	[95% Confidence Interval]	*p* Value
Age	1.03	0.98–1.09	0.222
Sex	1.16	0.36–3.71	0.803
Tear breakup time status Ϯ	1306.71	150.72–11329.29	<0.001
Schirmer’s I	0.91	0.70–1.19	0.512
MG grade	2.12	0.84–5.33	0.112

Ϯ Tear breakup time >5: coded as 1. Tear breakup time <5: coded as 2. Dependent variable: OSDI ≤ 32:0. OSDI > 32:1.

**Table 5:** Relationship of severe dry eye symptoms and tear breakup times (TBUT) in factory workers.

OSDI > 32	Overall	TBUT < 5 s	TBUT ≥ 5 s	*p* Value
**Overall**				
% (95%CI)	35.3 (32.4–38.3)	98.6 (96.9–99.6)	1.0 (0.3–1.4)	<0.001
[proportion]	[371/1050]	[364/369]	[7/681]	
**Sex**				
**Male**				
% (95%CI)	42.3 (37.8–46.8)	98.0 (95.1–99.5)	1.4 (0.4–3.6)	
[proportion]	[205/485]	[201/205]	[4/280]	<0.001
**Female**				
% (95%CI)	29.4 (25.7–33.3)	99.4 (96.7–99.98)	0.7 (0.1–0.8)	
[proportion]	[166/565]	[163/164]	[3/401]	<0.001
	*p* < 0.001			
**Age**				
**Younger(<35 years)**				
% (95%CI)	32.1 (27.8–36.6)	98.0 (94.2–99.6)	0.3 (0.0-1.8)	
[proportion]	[145/452]	[144/147]	[1/305]	<0.001
**Older(≥35 years)**				
% (95%CI)	37.8 (33.9–41.8)	99.1 (96.8–99.9)	1.6 (0.6–3.4)	
[proportion]	[226/598]	[220/222]	[6/376]	<0.001
	*p* = 0.059			

## References

[1] Craig J.P., Nichols K.K., Akpek E.K., Caffery B., Dua H.S., Joo C.K., Liu Z., Nelson J.D., Nicholas J.J., Tsubota K. (2017). TFOS DEWS II Definition and Classification Report. Ocul. Surf..

[2] Wolffsohn J.S., Arita R., Chalmers R., Djalilian A., Dogru M., Dumbleton K., Gupta P.K., Karpecki P., Lazreg S., Pult H. (2017). TFOS DEWS II Diagnostic Methodology report. Ocul. Surf..

[3] Craig J.P., Nelson J.D., Azar D.T., Belmonte C., Bron A.J., Chauhan S.K., de Paiva C.S., Gomes J., Hammitt K.M., Jones L. (2017). TFOS DEWS II Report Executive Summary. Ocul. Surf..

[4] Stapleton F., Alves M., Bunya V.Y., Jalbert I., Lekhanont K., Malet F., Na K.S., Schaumberg D., Uchino M., Vehof J. (2017). TFOS DEWS II Epidemiology Report. Ocul. Surf..

[5] Clegg J.P., Guest J.F., Lehman A., Smith A.F. (2006). The annual cost of dry eye syndrome in France, Germany, Italy, Spain, Sweden and the United Kingdom among patients managed by ophthalmologists. Ophthalmic Epidemiol..

[6] Wlodarczyk J., Fairchild C. (2009). United States cost-effectiveness study of two dry eye ophthalmic lubricants. Ophthalmic Epidemiol..

[7] Sullivan R.M., Cermak J.M., Papas A.S., Dana M.R., Sullivan D.A. (2002). Economic and quality of life impact of dry eye symptoms in women with Sjogren’s syndrome. Adv. Exp. Med. Biol..

[8] Yu J., Asche C.V., Fairchild C.J. (2011). The economic burden of dry eye disease in the United States: A decision tree analysis. Cornea.

[9] Reddy P., Grad O., Rajagopalan K. (2004). The economic burden of dry eye: A conceptual framework and preliminary assessment. Cornea.

[10] Chhadva P., Goldhardt R., Galor A. (2017). Meibomian Gland Disease: The Role of Gland Dysfunction in Dry Eye Disease. Ophthalmology.

[11] Nichols K.K., Foulks G.N., Bron A.J., Glasgow B.J., Dogru M., Tsubota K., Michael A.L., David A.S. (2011). The international workshop on meibomian gland dysfunction: Executive summary. Investig. Ophthalmol. Vis. Sci..

[12] Osae A.E., Gehlsen U., Horstmann J., Siebelmann S., Stern M.E., Kumah D.B., Steven P. (2017). Epidemiology of dry eye disease in Africa: The sparse information, gaps and opportunities. Ocul. Surf..

[13] Lee A.J., Lee J., Saw S.M., Gazzard G., Koh D., Widjaja D., Tan D.T. (2002). Prevalence and risk factors associated with dry eye symptoms: A population based study in Indonesia. Br. J. Ophthalmol..

[14] Shanti Y., Shehada R., Bakkar M.M., Qaddumi J. (2020). Prevalence and associated risk factors of dry eye disease in 16 northern West bank towns in Palestine: A cross-sectional study. BMC Ophthalmol..

[15] Sutradhar I., Gayen P., Hasan M., Gupta R.D., Roy T., Sarker M. (2019). Eye diseases: The neglected health condition among urban slum population of Dhaka, Bangladesh. BMC Ophthalmol..

[16] Lin P.Y., Tsai S.Y., Cheng C.Y., Liu J.H., Chou P., Hsu W.M. (2003). Prevalence of dry eye among an elderly Chinese population in Taiwan: The Shihpai Eye Study. Ophthalmology.

[17] Lin P.Y., Cheng C.Y., Hsu W.M., Tsai S.Y., Lin M.W., Liu J.H., Chou P. (2005). Association between symptoms and signs of dry eye among an elderly Chinese population in Taiwan: The Shihpai Eye Study. Investig. Ophthalmol. Vis. Sci..

[18] Chia E.M., Mitchell P., Rochtchina E., Lee A.J., Maroun R., Wang J.J. (2003). Prevalence and associations of dry eye syndrome in an older population: The Blue Mountains Eye Study. Clin. Exp. Ophthalmol..

[19] Uchino M., Dogru M., Yagi Y., Goto E., Tomita M., Kon T., Saiki M., Matsumoto Y., Uchino Y., Yokoi N. (2006). The features of dry eye disease in a Japanese elderly population. Optom. Vis. Sci..

[20] Hom M., De Land P. (2005). Prevalence and severity of symptomatic dry eyes in hispanics. Optom. Vis. Sci..

[21] Schaumberg D.A., Sullivan D.A., Buring J.E., Dana M.R. (2003). Prevalence of dry eye syndrome among US women. Am. J. Ophthalmol..

[22] Lin K.H., Su C.C., Chen Y.Y., Chu P.C. (2019). The effects of lighting problems on eye symptoms among cleanroom microscope workers. Int. J. Environ. Res. Public Health.

[23] Cho H.A., Cheon J.J., Lee J.S., Kim S.Y., Chang S.S. (2014). Prevalence of dry eye syndrome after a three-year exposure to a clean room. Ann. Occup. Environ. Med..

[24] Bulbulia A., Shaik R., Khan N., Vayej S., Kistnasamy B., Page T. (1995). Ocular health status of chemical industrial workers. Optom. Vis. Sci..

[25] Schiffman R.M., Christianson M.D., Jacobsen G., Hirsch J.D., Reis B.L. (2000). Reliability and validity of the Ocular Surface Disease Index. Arch. Ophthalmol..

[26] Asiedu K., Kyei S., Mensah S.N., Ocansey S., Abu L.S., Kyere E.A. (2016). Ocular surface disease index (OSDI) versus the standard patient evaluation of eye dryness (SPEED): A study of a nonclinical sample. Cornea.

[27] Vitale S., Goodman L.A., Reed G.F., Smith J.A. (2004). Comparison of the NEI-VFQ and OSDI questionnaires in patients with Sjogren’s syndrome-related dry eye. Health Qual. Life Outcomes.

[28] Özcura F., Aydin S., Helvaci M.R. (2007). Ocular surface disease index for the diagnosis of dry eye syndrome. Ocul. Immunol. Inflamm..

[29] Johnson M.E. (2009). The association between symptoms of discomfort and signs in dry eye. Ocul. Surf..

[30] Sullivan B.D., Crews L.A., Messmer E.M., Foulks G.N., Nichols K.K., Baenninger P., Geerling G., Figueiredo F., Lemp M.A. (2014). Correlations between commonly used objective signs and symptoms for the diagnosis of dry eye disease: Clinical implications. Acta Ophthalmol..

[31] Vehof J., Sillevis Smitt-Kamminga N., Nibourg S.A., Hammond C.J. (2017). Predictors of Discordance between Symptoms and Signs in Dry Eye Disease. Ophthalmology.

[32] Van Landingham S.W., West S.K., Akpek E.K., Muñoz B., Ramulu P.Y. (2014). Impact of dry eye on reading in a population-based sample of the elderly: The Salisbury Eye Evaluation. Br. J. Ophthalmol..

[33] Nichols K.K., Nichols J.J., Mitchell G.L. (2004). The lack of association between signs and symptoms in patients with dry eye disease. Cornea.

[34] Teo C.H.Y., Ong H.S., Liu Y.-C., Tong L. (2020). Meibomian gland dysfunction is the primary determinant of dry eye symptoms: Analysis of 2346 patients. Ocul. Surf..

[35] Tong L., Teo C.H.Y., Lee R.K.J. (2019). Spatial Distribution of Noninvasive Break Up Times and Clinical Relevance in Healthy Participants and Mild Dry Eye. Transl. Vis. Sci. Technol..

[36] Foong A.W., Saw S.M., Loo J.L., Shen S., Loon S.C., Rosman M., Aung T., Tan D.T., Tai E.S., Wong T.Y. (2007). Rationale and methodology for a population-based study of eye diseases in Malay people: The Singapore Malay eye study (SiMES). Ophthalmic Epidemiol..

[37] Koh S., Watanabe H., Hosohata J., Hori Y., Hibino S., Nishida K., Maeda N., Tano Y. (2003). Diagnosing dry eye using a blue-free barrier filter. Am. J. Ophthalmol..

[38] Peterson R.C., Wolffsohn J.S. (2009). Objective grading of the anterior eye. Optom. Vis. Sci..

[39] Terry R.L., Schnider C.M., Holden B.A., Cornish R., Grant T., Sweeney D., La Hood D., Back A. (1993). CCLRU standards for success of daily and extended wear contact lenses. Optom. Vis. Sci..

[40] Bruce A.S., Efron N. (2017). Preliminary Examination. Contact Lens Practice.

[41] Miller K.L., Walt J.G., Mink D.R., Satram-Hoang S., Wilson S.E., Perry H.D., Asbell P.A., Pflugfelder S.C. (2010). Minimal clinically important difference for the ocular surface disease index. Arch. Ophthalmol..

[42] Man R.E.K., Veerappan A.R., Tan S.P., Fenwick E.K., Sabanayagam C., Chua J., Leong Y.Y., Wong T.Y., Lamoureux E.L., Cheng C.Y. (2017). Incidence and risk factors of symptomatic dry eye disease in Asian Malays from the Singapore Malay Eye Study. Ocul. Surf..

[43] Jie Y., Xu L., Wu Y.Y., Jonas J.B. (2009). Prevalence of dry eye among adult Chinese in the Beijing Eye Study. Eye.

[44] Ranjan R., Shukla S.K., Veer Singh C., Mishra B.N., Sinha S., Sharma B.D. (2016). Prevalence of Dry Eye and Its Association with Various Risk Factors in Rural Setup of Western Uttar Pradesh in a Tertiary Care Hospital. Open J. Prev. Med..

[45] Song H., Zhang M., Hu X., Li K., Jiang X., Liu Y., Lv H., Li X. (2017). Correlation Analysis of Ocular Symptoms and Signs in Patients with Dry Eye. J. Ophthalmol..

[46] Shimazaki J., Sakata M., Tsubota K. (1995). Ocular Surface Changes and Discomfort in Patients With Meibomian Gland Dysfunction. Arch. Ophthalmol..

[47] Fu J., Chou Y., Hao R., Jiang X., Liu Y., Li X. (2019). Evaluation of ocular surface impairment in meibomian gland dysfunction of varying severity using a comprehensive grading scale. Medicine.

[48] Chao C., Tong L. (2018). Tear lactoferrin and features of ocular allergy in different severities of meibomian gland dysfunction. Optom. Vis. Sci..

[49] Sugiura K., Sugiura M., Hayakawa R., Shamoto M., Sasaki K. (2008). A case of contact urticaria syndrome due to di(2-ethylhexyl) phthalate (DOP) in work clothes. Contact Dermat..

[50] Iadaresta F., Manniello M.D., Östman C., Crescenzi C., Holmbäck J., Russo P. (2018). Chemicals from textiles to skin: An in vitro permeation study of benzothiazole. Environ. Sci. Pollut. Res..

[51] Tsuboy M.S., Angeli J.P.F., Mantovani M.S., Knasmüller S., Umbuzeiro G.A., Ribeiro L.R. (2007). Genotoxic, mutagenic and cytotoxic effects of the commercial dye CI Disperse Blue 291 in the human hepatic cell line HepG2. Toxicol. In Vitro.

[52] (2014). Chemicals in Textiles-Risks to Human Health and the Environment.

[53] Jung S.J., Mehta J.S., Tong L. (2018). Effects of environment pollution on the ocular surface. Ocul. Surf..

[54] Ambaw Y.A., Chao C., Ji S., Raida M., Torta F., Wenk M.R., Tong L. (2018). Tear eicosanoids in healthy people and ocular surface disease. Sci. Rep..

[55] Lam S.M., Tong L., Reux B., Duan X., Petznick A., Yong S.S., Khee C.B., Lear M.J., Wenk M.R., Shui G. (2014). Lipidomic analysis of human tear fl uid reveals structure-specific lipid alterations in dry eye syndrome. J. Lipid Res..

[56] Lam S.M., Tong L., Yong S.S., Li B., Chaurasia S.S., Shui G., Wenk M.R. (2011). Meibum lipid composition in Asians with dry eye disease. PLoS ONE.

[57] Tong L., Zhou L., Beuerman R.W., Zhao S.Z., Li X.R. (2011). Association of tear proteins with Meibomian gland disease and dry eye symptoms. Br. J. Ophthalmol..

[58] Yolton D.P., Mende S., Harper A., Softing A. (1991). Association of dry eye signs and symptoms with tear lactoferrin concentration. J. Am. Optom. Assoc..

[59] Lam H., Bleiden L., de Paiva C.S., Farley W., Stern M.E., Pflugfelder S.C. (2009). Tear Cytokine Profiles in Dysfunctional Tear Syndrome. Am. J. Ophthalmol..

[60] Napoli P.E., Nioi M., Mangoni L., Gentile P., Braghiroli M., d’Aloja E., Fossarello M. (2020). Fourier-Domain OCT Imaging of the Ocular Surface and Tear Film Dynamics: A Review of the State of the Art and an Integrative Model of the Tear Behavior During the Inter-Blink Period and Visual Fixation. J. Clin. Med..

[61] Ahmed S., Taher I., Ghoneim D., Safwat A. (2019). Effect of intense pulsed light therapy on tear proteins and lipids in meibomian gland dysfunction. J. Ophthalmic Vis. Res..

[62] Yin Y., Liu N., Gong L., Song N. (2018). Changes in the Meibomian Gland After Exposure to Intense Pulsed Light in Meibomian Gland Dysfunction (MGD) Patients. Curr. Eye Res..

[63] Arita R., Mizoguchi T., Fukuoka S., Morishige N. (2018). Multicenter Study of Intense Pulsed Light Therapy for Patients with Refractory Meibomian Gland Dysfunction. Cornea.

[64] Rong B., Tang Y., Liu R., Tu P., Qiao J., Song W., Yan X. (2018). Long-Term Effects of Intense Pulsed Light Combined with Meibomian Gland Expression in the Treatment of Meibomian Gland Dysfunction. Photomed. Laser Surg..

